# Elevated GAS2L3 Expression Correlates With Poor Prognosis in Patients With Glioma: A Study Based on Bioinformatics and Immunohistochemical Analysis

**DOI:** 10.3389/fgene.2021.649270

**Published:** 2021-03-30

**Authors:** Yan Zhou, Limin Zhang, Sirong Song, Lixia Xu, Yan Yan, Haiyang Wu, Xiaoguang Tong, Hua Yan

**Affiliations:** ^1^Clinical College of Neurology, Neurosurgery and Neurorehabilitation, Tianjin Medical University, Tianjin, China; ^2^Tianjin Key Laboratory of Cerebral Vascular and Neurodegenerative Diseases, Tianjin Neurosurgical Institute, Tianjin Huanhu Hospital, Tianjin, China; ^3^Department of Clinical Laboratory, Tianjin Huanhu Hospital, Tianjin, China; ^4^Department of Neurosurgery, Tianjin Huanhu Hospital, Tianjin, China

**Keywords:** GAS2L3, glioma, bioinformatics, biomarker, tumor microenvironment

## Abstract

**Background:**

Growth arrest–specific 2 like 3 (GAS2L3) is a cytoskeleton-associated protein that interacts with actin filaments and tubulin. Abnormal GAS2L3 expression has been reported to be associated with carcinogenesis. However, the biological role of GAS2L3 in glioma remains to be determined.

**Methods:**

The transcriptome level of GAS2L3 and its relationship with clinicopathological characteristics were analyzed among multiple public databases and clinical specimens. Bioinformatics analyses were conducted to explore biological functions and prognostic value of GAS2L3 in glioma.

**Results:**

GAS2L3 was substantially expressed in glioma, and high GAS2L3 expression correlated with shorter overall survival time and poor clinical variables. Gene set enrichment analysis (GSEA), single-sample gene-set enrichment analysis, and CIBERSORT algorithm analyses showed that GAS2L3 expression was closely linked to immune-related pathways, inflammatory activities, and immune cell infiltration. Moreover, GAS2L3 was synergistic with T cell–inflamed gene signature, immune checkpoints, T-cell receptor diversities, and neoantigen numbers.

**Conclusion:**

This study suggests that GAS2L3 is a prognostic biomarker for glioma, providing a reference for further study of the potential role of GAS2L3 in the immunomodulation of glioma.

## Introduction

Worldwide, gliomas account for approximately 40–50% of all neoplasms of the central nervous system (CNS) (Ostrom et al., 2018). Although advances in glioma treatment have been achieved in the past decades, the therapeutic efficacy, especially on glioblastoma (GBM), is still limited (5-year survival are approximately only 5.5%) (Omuro and DeAngelis, 2013). The molecular mechanisms underlying tumor development and progression are poorly understood, and the lack of specific markers for tumor type or disease stage further impedes the current understanding and treatments of glioma.

The growth arrest–specific 2 (GAS2) family, consisting of four related proteins (GAS2, GAS2L1, GAS2L2, and GAS2L3), participate in cross-linking of actin and microtubule filaments in interphase and in growth-arrested cells (Goriounov et al., 2003; Stroud et al., 2011). Unlike members, GAS2L3 mRNA expression in resting cells is extremely low, whereas the expression level gradually increases when the cell reenters the cell cycle and reaches a peak in the G2/M phase (Wolter et al., 2012). The loss of GAS2L3 and overexpression studies have implicated GAS2L3 in cytokinesis, chromosome segregation, and abscission (Pe’Er et al., 2013). Recently, it has been reported that GAS2L3 was dysregulated in various tumor cells. Shi et al. (2020) reported that GAS2L3 was significantly related to the deterioration of overall survival (OS) and disease-free survival in hepatocellular carcinoma. Seidl et al. (2010) reported that GAS2L3 was downregulated after incubation with highly cytotoxic α-emitter immunoconjugates in gastric cancer cells. However, the role of GAS2L3 in glioma has not been reported.

Here, we investigated the association between GAS2L3 gene expression and glioma clinical characteristics using data from public databases and clinical samples from our institution. Additionally, its potential roles in immune response and immune infiltration of tumor microenvironment (TME) were analyzed. Our results could potentially reveal new targets and strategies for glioma diagnosis and treatment.

## Materials and Methods

### Dataset Selection

The mRNA level of GAS2L3 in different cancer types was confirmed by the Oncomine database. In glioma, gene expression and relevant clinical data of patients were obtained from five cancer datasets. The Cancer Genome Atlas (TCGA) (Wang et al., 2016) dataset was used as a discover set, whereas the Chinese Glioma Genome Atlas (CGGA) (Yan et al., 2012), the GSE16011 (from Gene Expression Omnibus database) (Gravendeel et al., 2009), and the Repository of Molecular Brain Neoplasia Data (REMBRANDT) (Madhavan et al., 2009) as validation sets. Additionally, we enrolled a total of 127 glioma patients from the department of neurosurgery, Huanhu Hospital (Tianjin, China), as the external validation set. All patients signed an informed consent form. The study protocol was approved by Huanhu Hospital Ethics Committee (Tianjin, China).

### Immunohistochemistry

Tumor tissues were surgically excised and were immediately fixed in 10% neutral buffered formalin for 24 h and then embedded in paraffin. Tissue slides were prepared and deparaffinized by baking in oven 60°C for 1 h. Then, antigen unmasking was done in boiling container with sodium citrate buffer for 20 min. Goat serum was used for blocking. Slides were then stained with GAS2L3 rabbit polyclonal antibody (1:400, bioss, BS-23297R) overnight at 4°C, and secondary antibodies were incubated for 1 h at room temperature. Positive or negative staining of GAS2L3 was independently evaluated by two experienced pathologists, and samples were divided into two groups: low expression group, including negative (−) and weak (+) staining, and high expression group, including moderate (+ +) and strong (+ + +) staining (Xiao et al., 2015).

### Bioinformatics Analysis

The examination of tumor/normal differential expression analysis of GAS2L3 in TCGA was performed by GEPIA, an interactive web server containing 8,587 normal samples from the GTEx database (Tang et al., 2019). The correlation between GAS2L3 expression and various clinical characteristics was analyzed and plotted using beeswarm R package. Kaplan--Meier curve, receiver operating characteristic (ROC) curve, and area under the ROC curve (AUC) were graphed using survival, survminer, and ROC packages. Univariate and multivariate Cox analyses were performed to compare the impact of GAS2L3 expression on the OS alongside with other clinical variables. The bioinformatics analyses were performed utilizing R software v3.6.3. All R packages were downloaded from CRAN^1^ and Bioconductor^2^.

### Meta-Analysis

We performed a meta-analysis to assess the overall prognostic value of GAS2L3 in glioma patients among four datasets. Combined hazard ratio (HR) and 95% confidence interval (CI) were used to measure the effect size. The heterogeneity among datasets was assessed by the *Q* test (*I*^2^ statistics). The random-effects model was used to minimize the influence of heterogeneity, whereas *I*^2^ > 50% or *P* < 0.10; otherwise, the fixed-effects model would be applied. The meta-analysis was conducted in STATA 15 software.

### Comprehensive Correlation Analysis in Tumor Immunity

In the discovery cohort, gene set enrichment analysis (GSEA) (Subramanian et al., 2005) was conducted to identify Gene Ontology (GO) and Kyoto Encyclopedia of Genes and Genomes (KEGG) pathways, which showed statistically significant differences between high and low GAS2L3 expression cohorts. The annotated gene sets of c5.all.v7.2.symbols.gmt and c2.cp.kegg.v7.2.symbols.gmt in the MSigDB were selected in GSEA version 4.0 software. The false discovery rate < 0.25 and normal *P* < 0.05 were used as thresholds. The single-sample GSEA (ssGSEA) was used to analyze the RNA-seq data of 29 important immune signatures from each glioma sample in the form of ssGSEA scores. The signatures for immune cell types were obtained from previous publications (Bindea et al., 2013; Liu et al., 2018; Zhou et al., 2020). Furthermore, 22 types of tumor-infiltrating immune cells [tumor-infiltrating lymphocytes (TILs)] in glioma microenvironment were assessed based on CIBERSORT deconvolution algorithm (Newman et al., 2015). The gene expression signature of 22 TILs was obtained from the CIBERSORT platform^3^.

The T cell–inflamed gene signature, obtained from a previously report (Ayers et al., 2017), was calculated by gene set variation analysis (GSVA). Then, we calculated the expression levels of programmed cell death ligand 1 (PD-L1) and other immune checkpoints in sample with GAS2L3-low and -high. Gene indel mutations and single-nucleotide variants are prone to result in major histocompatibility complex–binding neoantigens, which can be recognized by immune cells. The neoantigen load for each patient from the discovery cohort was obtained from a previous paper (Schumacher and Schreiber, 2015). The T-cell receptor (TCR) diversities of patients from the discovery cohort, which were measured using the Shannon entropy, were obtained from a previous report (Thorsson et al., 2018).

### Statistical Analysis

Patients with missing information were excluded from the corresponding analysis. Mann–Whitney *U* and Kruskal–Wallis test were used to analyze the relationship between GAS2L3 mRNA expression and the clinical features of glioma. For GAS2L3 protein expression, χ^2^ test was employed. The proportions of TILs between GAS2L3 expression-low and -high subtypes were compared using the Mann–Whitney *U* test. The statistical analyses were performed utilizing R software v3.6.3 and GraphPad Prism 7. All statistical tests were two-sided. *P* < 0.05 was used to determine the significance level.

## Results

### Preparation of Datasets

A total of 2,604 cases (including 41 non-tumor samples) from discovery and validation datasets were included in this study. The characteristics of the glioma patients in these five datasets were concluded in Table 1. Patients’ detailed information of Huanhu dataset is shown in Supplementary Table 1.

**TABLE 1:** Characteristics of patients in TCGA, CGGA, GSE16011, REMBRANDT, and Huanhu datasets.

Characteristic	TCGA	CGGA	GSE16011	REMBRANDT	Huanhu
**Total**	703	1,018	284	472	127
**Age (years)**					
≥52	263	191	133		45
<52	407	558	143		82
**Gender**					
Male	651	442	184	221	77
Female	460	307	92	126	50
**Grade**					
I			8	2	
II	249	218	24	98	39
III	265	240	85	85	35
IV	596	291	159	130	53
**Histology**					
Pilocytic astrocytoma			8		
Astrocytoma	194	150	29	147	28
Oligodendroglioma	191	76	52	67	46
Oligoastrocytoma	130	232	28		
Glioblastoma	596	291	159	219	53
Mixed glioma				11	
**IDH1 mutation**					
Yes	91	410	81		87
No	34	339	140		40
**1p19q codeleted**					
Yes		155	110		
No		594	45		
**KPS**					
<80	151		82		
≥80	584		182		

### GAS2L3 Transcript Levels in Different Databases

**FIGURE 1:**
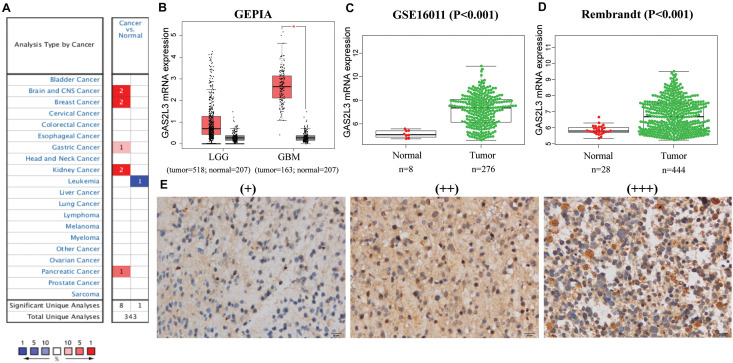
The mRNA and protein expression levels of GAS2L3. **(A)** GAS2L3 expression level in cancers in Oncomine database: the left box in red indicates the number of datasets with GAS2L3 overexpression, and the right box in blue indicates the number of datasets with GAS2L3 hypoexpression after comparing cancerous and normal tissues. **(B–D)** TCGA (based on GEPIA), GSE16011, and REMBRANDT datasets support the findings that indicate GAS2L3 upregulation in glioma (*P < 0.05). **(E)** GAS2L3 protein expression was detected in glioma tissues from Huanhu dataset.

First, the mRNA levels of GAS2L3 in different cancers were analyzed in Oncomine database. Relative to control specimens, GAS2L3 was significantly upregulated in brain and CNS, breast cancer, gastric cancer, kidney cancer, and pancreatic cancer, but downregulated in leukemia (Figure 1A). These results suggest that the high expression of GAS2L3 is common in various types of cancer.

In glioma, GAS2L3 mRNA expression data from TCGA and 207 normal samples from the GTEx project were analyzed based on GEPIA, and we found that GAS2L3 was significantly upregulated in GBM and had a relative increased expression in lower-grade glioma compared to normal samples (Figure 1B). The differential expression was also confirmed in validation datasets (Figures 1C,D). Additionally, the expression levels of other GAS2 members in glioma were also analyzed by GEPIA, but no upregulation was found compared with the control group (Supplementary Figure 1).

For protein expression level, immunohistochemistry (IHC) staining indicated that 67.7% (86/127) of glioma tissues had high GAS2L3 expression. Representative slides are displayed in Figure 1E and Supplementary Figure 1. Taken together, GAS2L3 was highly expressed at both transcriptional and proteomic levels in glioma tissues.

### GAS2L3 Is Associated With Clinicopathological Features of Glioma

**FIGURE 2:**
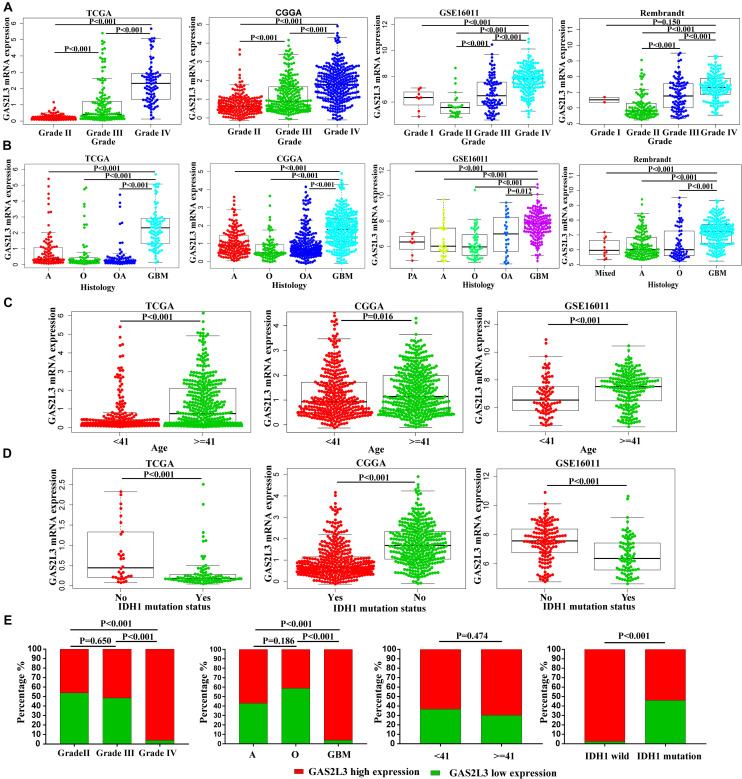
Associations between GAS2L3 expression and clinicopathologic variables in different datasets. **(A)** WHO grade, **(B)** histological type; PA: pilocytic astrocytoma, A: astrocytoma, O: oligodendroglioma, OA: oligoastrocytoma, mixed: mixed histological type, GBM: glioblastoma, **(C)** age (years), **(D)** IDH1 mutation status, **(E)** the differential protein expression of GAS2L3 in samples from Huanhu dataset by IHC method.

Because of the heterogeneity of glioma (Lapointe et al., 2018), the mRNA expression data were analyzed according to the World Health Organization (WHO) grade, histology, age, isocitrate dehydrogenase 1 (IDH1) mutation, and other features. In TCGA, CGGA, GSE16011, and Rembrandt datasets, the expression of GAS2L3 was the highest in the grade IV compared with the low-grade tumors (Figure 2A). Besides, GAS2L3 expression increased in higher histopathologic malignancies (Figure 2B). As for age and IDH1 mutation status, the higher expression level of GAS2L3 was detected in patients older than 41 years and those with wild-type IDH1 in TCGA, CGGA, and GSE16011 datasets (Figures 2C,D). Then, we quantified the protein level of GAS2L3 in Huanhu cohort by IHC staining. GAS2L3 was highly expressed in advanced grades and histopathologic types and wild-type IDH1, although no association was found between GAS2L3 expression and age (Figure 2E). Additionally, some other clinical characteristics [1p19q codeletion status, Karnofsky Performance Status (KPS), chemotherapy, radiotherapy, gender and recurrent status] were also analyzed (Supplementary Figure 2). These results indicated that high expression of GAS2L3 predicted high malignant glioma.

### GAS2L3 Predicts Worse Survival in Glioma

**FIGURE 3:**
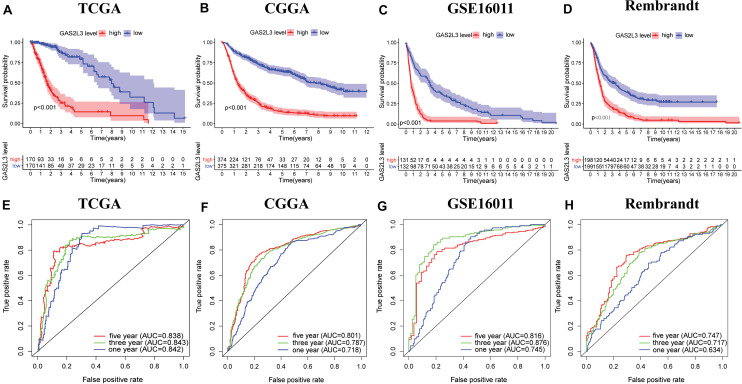
Associations between GAS2L3 expression and prognosis of patients in different datasets. **(A–D)** Kaplan–Meier estimated of overall survival between groups harboring different GAS2L3 expression. **(E–H)** Time-dependent ROC curves of overall survival at 1, 3, and 5 years.

To investigate the prognostic value of GAS2L3 expression, patients were divided into low or high groups based on the median expression value. Kaplan–Meier plots demonstrated that high level of GAS2L3 expression was correlated with unfavorable OS of glioma patients in different datasets (Figures 3A–D). ROC analysis showed that GAS2L3 could be a good predictor of 1-year (AUC = 0.842), 3-year (AUC = 0.843), and 5-year survival (AUC = 0.838) (Figure 3E). These results were validated in other datasets (Figures 3F–H).

### Cox Regression Analysis and Meta-Analysis

**FIGURE 4:**
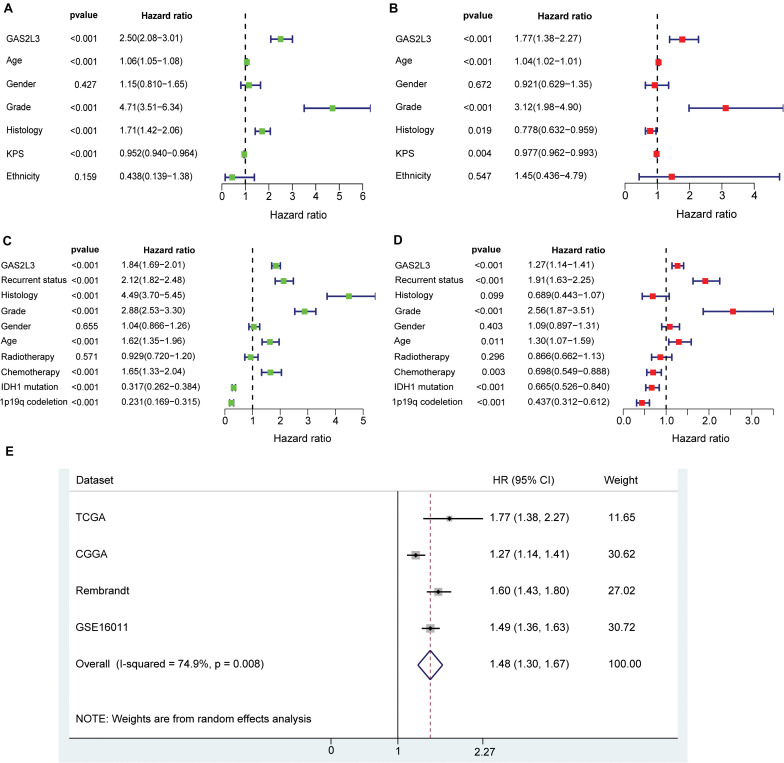
Univariate and multivariate Cox regression analyses regarding OS in TCGA dataset **(A,B)** and CGGA validation dataset **(C,D)**. Precise forest plots were graphed, respectively. **(E)** Forest plot of a meta-analysis of high GAS2L3 expression with worse OS in glioma patients from four datasets.

To further explore the prognostic value of GAS2L3, univariate and multivariate Cox regression analyses were conducted in both TCGA and CGGA datasets. In TCGA cohort, the univariate analysis showed that patients with GAS2L3-high expression had worse OS (HR = 2.50, 95% CI [2.08–3.01], *P* < 0.001) (Figure 4A). Besides, clinical characteristics, age (HR = 1.06, 95% CI [1.05–1.08]), grade (HR = 4.71, 95% CI [3.51–6.34]), histological type (HR = 1.71, 95% CI[1.42–2.06]), and KPS (HR = 0.952, 95% CI [0.940–0.964]) also correlated significantly with poor survival (all with *P* < 0.001). After adjusting for other clinicopathologic characteristics, the multivariate analysis revealed that GAS2L3 expression remained independently associated with OS (Figure 4B, HR = 1.77, 95% CI [1.38–2.27], *P* < 0.001). These results were also validated in CGGA cohort (Figures 4C,D), demonstrating that GAS2L3 expression is an independent prognostic factor of glioma.

As no published studies have focused on the prognostic role of GAS2L3 expression in glioma, an integrated meta-analysis of four datasets was carried out to assess the overall prognostic value of GAS2L3 in glioma patients (Figure 4E). The pooled HR (HR = 1.48, 95% CI [1.30–1.67]) using the random-effects model suggested that a higher expression level of GAS2L3 significantly predicted poorer OS in patients with glioma. However, heterogeneity was found (*I*^2^ = 74.9%, *P* = 0.008), which may be partly attributed to the differences of Cox regression analyses for HR, sequencing methods, or countries among patients in four datasets.

### GAS2L3-Related Immune and Inflammatory Pathways

**FIGURE 5:**
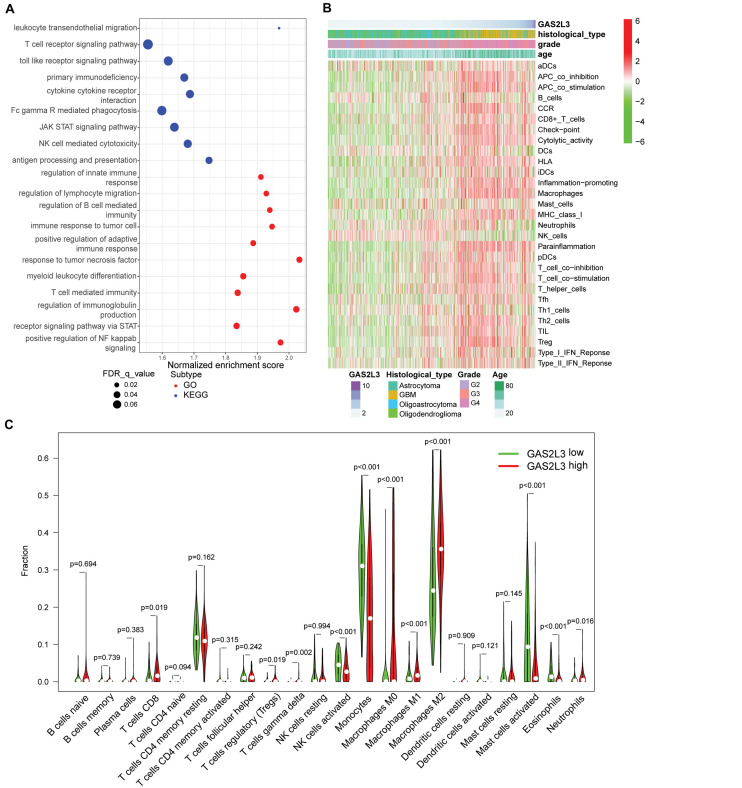
GAS2L3-related inflammatory activities and immune infiltration in TCGA. **(A)** GO and KEGG pathway analysis. **(B)** Heatmaps displaying GAS2L3 expression, clinicopathological parameters, and 29 well-established immune-associated gene sets based on ssGSEA method. **(C)** The different proportions of 22 TILs in glioma samples based on CIBERSORT algorithm.

Patients with high GAS2L3 expression had a short OS time, suggesting that GAS2L3 may be involved in the initiation and the progression of glioma. Then, we performed GSEA to identify GO and KEGG signaling pathways, which were enriched in GAS2L3-high expression phenotype in TCGA. The GO analysis showed that a set of pathways, especially those related to immunity, was enriched, including regulation of innate immune response, regulation of lymphocyte migration, regulation of B cell–mediated immunity, immune response to tumor cell, and so on. Meanwhile, immune-related pathways, such as leukocyte transendothelial migration, TCR signaling pathway, and toll-like receptor signaling pathway, were also enriched in a cohort with GAS2L3-high by KEGG analysis (Figure 5A). These results indicated that GAS2L3 may play an essential role in the immune microenvironment of glioma.

### The Relationship Between GAS2L3 and Immune Infiltration and Activities

To better comprehend the role of GAS2L3 in immune activities, we used the ssGSEA to explore the relationship between GAS2L3 and activities or enrichment levels of immune cells and functions, based on the 29 well-established immune-associated gene sets (Bindea et al., 2013; Liu et al., 2018; Zhou et al., 2020). As the heatmap showed (Figure 5B), some immune cell types, such as CD8^+^ T, macrophages, T helper cells, regulatory T cells (Tregs), immature dendritic cells (iDCs), and plasmacytoid dendritic cells (pDCs), were infiltrated in glioma samples with GAS2L3-high, whereas natural killer cells (NK cells) had an opposite trend. Additionally, some immune-associated functions also had positive correlations with GAS2L3 expression. The marker genes of antigen-presenting cells (APCs) costimulation, APC coinhibition, chemokine receptor (CCR), immune checkpoint, cytolytic activity, human leukocyte antigen, inflammation promoting, major histocompatibility complex class I, parainflammation, T-cell costimulation, T-cell coinhibition, and types I and II interferon (IFN) responses were more highly expressed in samples with GAS2L3-high than in those with GAS2L3-low.

As TILs play an essential pathophysiological role in the development of glioma (Domingues et al., 2016), we systematically estimated the proportions of 22 TILs in TCGA glioma samples based on CIBERSORT algorithm (Newman et al., 2015). The results showed the TIL subsets had significantly different proportions in different GAS2L3 expression cohorts (Figure 5C). Coincided with ssGSEA analysis, we observed that CD8^+^ T cells, Tregs, *γδ* T cells, neutrophils, and macrophages M0, M1, and M2 were enriched in GAS2L3-high cohort. Nevertheless, monocytes, NK cells activated, mast cells activated, and eosinophils were enriched in the GAS2L3-low cohort. These results indicated that GAS2L3 has a close relationship with immunomodulation of glioma.

### GAS2L3 Is a Predictive Marker for Response of Immunotherapy

**FIGURE 6:**
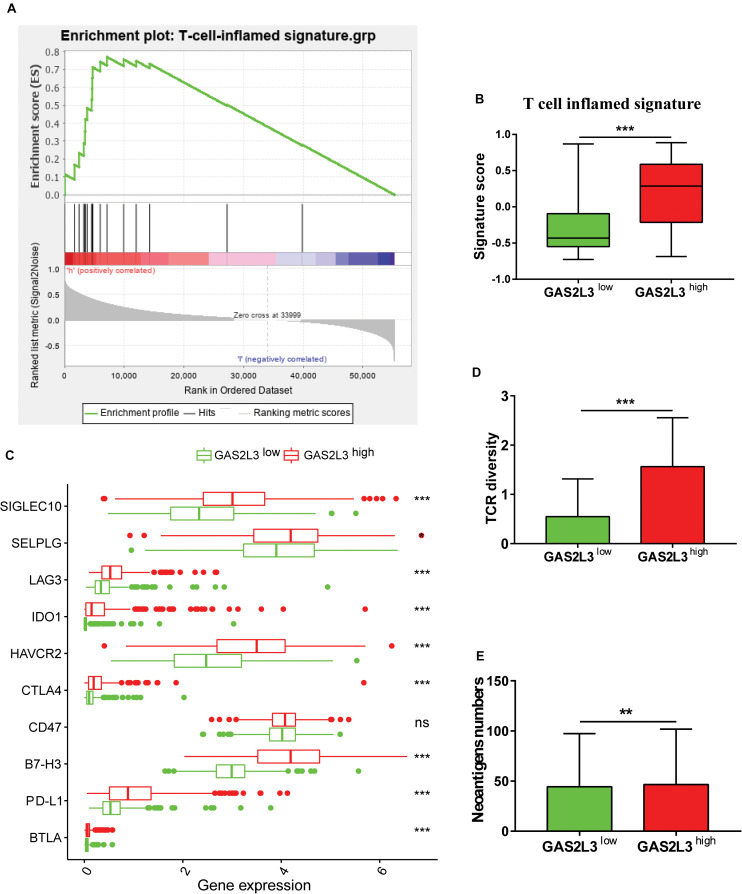
GAS2L3-high and -low tumors exhibit features of anti–PD-1 responsiveness. **(A)** GSEA of the T-cell–inflamed gene signature in GAS2L3-high and -low tumors. **(B)** T-cell–inflamed gene signature scores in GAS2L3-high and -low groups. **(C)** GAS2L3 was synergistic with PD-L1 and other immune checkpoints. **(D)** T-cell receptor (TCR) diversity in GAS2L3-high and -low tumors. **(E)** Neoantigen numbers in GAS2L3-high and -low tumors. ns, not significant (*P < 0.05, **P < 0.01, ***P < 0.001).

Traditional therapies combined with immunotherapies, such as immune checkpoint inhibitors and chimeric antigen receptor T cells, have gained promising results in multiple tumor treatments (Abril-Rodriguez and Ribas, 2017). Several studies have explored the usage of immunotherapy against glioma (Suryadevara et al., 2015); however, the therapeutic efficacy was still less than satisfactory. Here, we investigated the potential of patients with different GAS2L3 expression to respond to anti–programmed cell death 1 (PD-1) therapy. As Figure 6A showed, the T cell–inflamed signature, a predictor of responses to anti–PD-1 therapy in various types of cancer (Ayers et al., 2017), was significantly enriched in tumors with GAS2L3-high based on GSEA analysis. The T cell–inflamed signature score calculated by GSVA was also higher in GAS2L3-high tumors (Figure 6B). Patients with higher PD-L1 expression tend to gain more clinical benefits from anti–PD-1 therapy (Ayers et al., 2017). Here, we found that GAS2L3 had positive associations with PD-L1, B7-H3, and other immune checkpoints (Figure 6C). The TCR diversity and neoantigen numbers, also known to be predictive markers for anti–PD-1 therapy (Tumeh et al., 2014), were similarly higher in tumors with GAS2L3-high (Figures 6D,E). In conclusion, GAS2L3 can help to predict response of antitumor immunotherapy.

## Discussion

Glioma is the most prevalent and lethal primary brain tumors in adults (Ostrom et al., 2018). Because of the limited improvements in the treatment of glioma, new therapeutic methods are urgently needed. A variety of indicators, such as genetic aberrations and tumor environment, have been reported to participate in the development and progression of glioma (Molinaro et al., 2019). The potential roles of GAS2 family in tumorigenesis of liver cancer (Zhu et al., 2015), leukemia (Kong et al., 2020), recurrent colorectal cancer (Chang et al., 2016), or lung adenocarcinoma (Murugesan et al., 2018) have been explored. However, the prognostic value of GAS2L3 in glioma still remains unclear. In this study, we evaluated the expression level and prognostic value of GAS2L3 in glioma based on public databases and clinical specimens. We observed that GAS2L3 was significantly upregulated in glioma that was associated with malignant behavior. Our Cox regression models showed that a high level of GAS2L3 expression correlated with shorter patients’ survival time. Meta-analysis containing patients from four public databases further established the critical role of high GAS2L3 expression in the adverse prognosis of glioma patients. Functional enrichment analysis illustrated that GAS2L3 was significantly involved in plenty of immune-related pathways, such as regulation of lymphocyte migration, TCR signaling pathway, and immune response to tumor cell.

Studies have shown that the TME, especially immune microenvironment, has a great impact on the development of cancers (Li et al., 2017; Wang et al., 2017). However, the functions of GAS2L3 in the TME have not been reported. Here, we employed two different methods (ssGSEA analysis and CIBERSORT algorithm) to investigate the impact of GAS2L3 on infiltration of immune cells in glioma. ssGSEA showed that GAS2L3 was positively correlated with the infiltration of various immune cell types, including T cells [CD8^+^ T cells, T helper cell 1 [T_*H*_1] cells, T_*H*_2 cells, PD-L1], macrophages, iDCs, and pDCs, whereas NK cells had an opposite trend. CIBERSORT algorithm further revealed that GAS2L3 impacted the proportions of 22 TILs in glioma. Patients with different level of GAS2L3 expression had significant differences in the proportion of immune infiltration. For example, the proportions of CD8^+^ T cells, Tregs, *γδ* T cells, neutrophils, and macrophages M0, M1, and M2 were higher in the group with GAS2L3-high, which were coincided with the ssGSEA analysis. The infiltration of DCs, the classical APCs, can help present tumor-associated antigens to T cells (Gardner and Ruffell, 2016). The increase of CD8^+^ T cells can secrete various cytokines and generate cytolytic activity to enhance the antitumor immunity (Andre et al., 2018). The priming of CD8^+^ cytotoxic T lymphocytes generally requires the participation of CD4^+^ T-helper lymphocytes (Schoenberger et al., 1998). GAS2L3 also upregulated the infiltration of Tregs, which maintain a balance to fight diseases and at the same time prevent damage to healthy tissues (Schoenberger et al., 1998; Maghazachi, 2003). It has been reported that macrophages have a double effect on the development of tumor (Wang et al., 2015). M1 macrophages participate in antigen presentation and immune surveillance by secreting proinflammatory chemokines, whereas M2 macrophages exert inhibitory function (Chen et al., 2019). Through the ssGSEA analysis, we also found GAS2L3 may upregulate the level of CCRs and IFN (both types I and II), which are critical for immune cell recruitment and CD8^+^ T cell activation (Balkwill, 2004; Farhood et al., 2019). Consequently, GAS2L3 may play a critical role in regulating TME in glioma by participating in cellular and humoral immunity.

In the last decade, immune checkpoint inhibitors have shown remarkable success in treating various tumors (Abril-Rodriguez and Ribas, 2017). However, because of the substantial heterogeneity of tumor cells, the efficacy of immunotherapies was less than satisfactory in glioma (Suryadevara et al., 2015). Identifying those who may respond to immunotherapies will certainly help glioma patients gain more clinical benefits from anti–PD-1 therapy. In this study, we employed several predictive markers for anti–PD-1 therapy, which have been discussed in multiple types of cancer. We found that the T cell–inflamed signature, PD-L1 expression, the TCR diversity, and neoantigen numbers were significantly enriched in tumors with GAS2L3-high. In conclusion, it makes sense that GAS2L3 in combination with these biomarkers may help identify patients who have a higher likelihood of response to immunotherapies.

The current study had some limitations. First, heterogeneity was found in our meta-analysis, and we could hardly explain its source. Thus, more clinical research projects are needed for further elucidation. Second, the mechanisms by which GAS2L3 may play an important role in the immunomodulation of the glioma microenvironment need further experimental verification. Third, more data are needed to prove the efficacy of the combination of GAS2L3 target and immunotherapies.

In summary, multicenter data showed that GAS2L3 was upregulated in advanced glioma and was related to adverse clinical outcomes. We found that GAS2L3 was involved in numerous immune activities and immune cell infiltration. It makes sense to combine anti-GAS2L3 and anti–PD-1 therapies to amplify the efficacy of treatments typically used in isolation. Taken together, GAS2L3 may act as a potential biomarker of prognosis and therapeutic target for glioma.

## Data Availability Statement

The original contributions presented in the study are included in the article/Supplementary Material, further inquiries can be directed to the corresponding authors.

## Ethics Statement

All patients signed an informed consent form. The study protocol was approved by Tianjin Huanhu Hospital Ethics Committee (Tianjin, China).

## Author Contributions

YZ, LX, and HY: study design. YZ, LZ, SS, and HW: data collection. YZ, LZ, YY, and HW: data analysis and interpretation. YZ, HW, XT, and HY: writing, review, polishing, and revision of the manuscript.

## Conflict of Interest

The authors declare that the research was conducted in the absence of any commercial or financial relationships that could be construed as a potential conflict of interest.

## Footnotes

1 http://cran.r-project.org/

2 http://bioconductor.org/

3 https://cibersortx.stanford.edu/
