# Supplementary material for: Elevated GAS2L3 Expression Correlates With Poor Prognosis in Patients With Glioma: A Study Based on Bioinformatics and Immunohistochemical Analysis
Source: Front Genet. 2021 Mar 30;12:649270. doi: 10.3389/fgene.2021.649270 (PMC8042292; doi:10.3389/fgene.2021.649270)
Supplement: Supplementary file 1 [file Data_Sheet_1.docx]

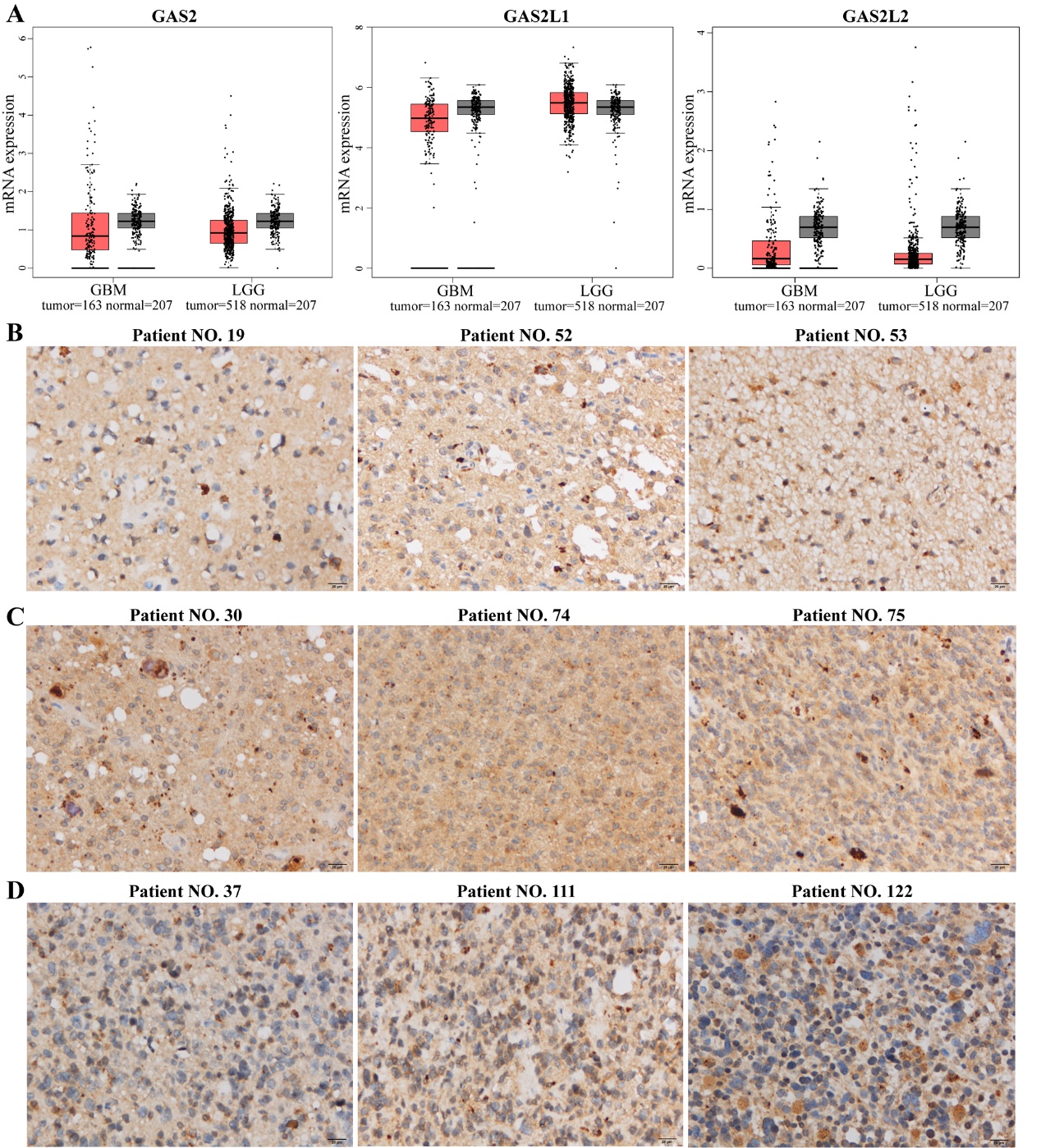


**Figure S1.** (A) The mRNA expression levels of other GAS2 family members in GEPIA. (B, C and D) Representative IHC images of samples with grade Ⅱ, Ⅲ and IV, respectively. Patient numbers were included in Table S1.


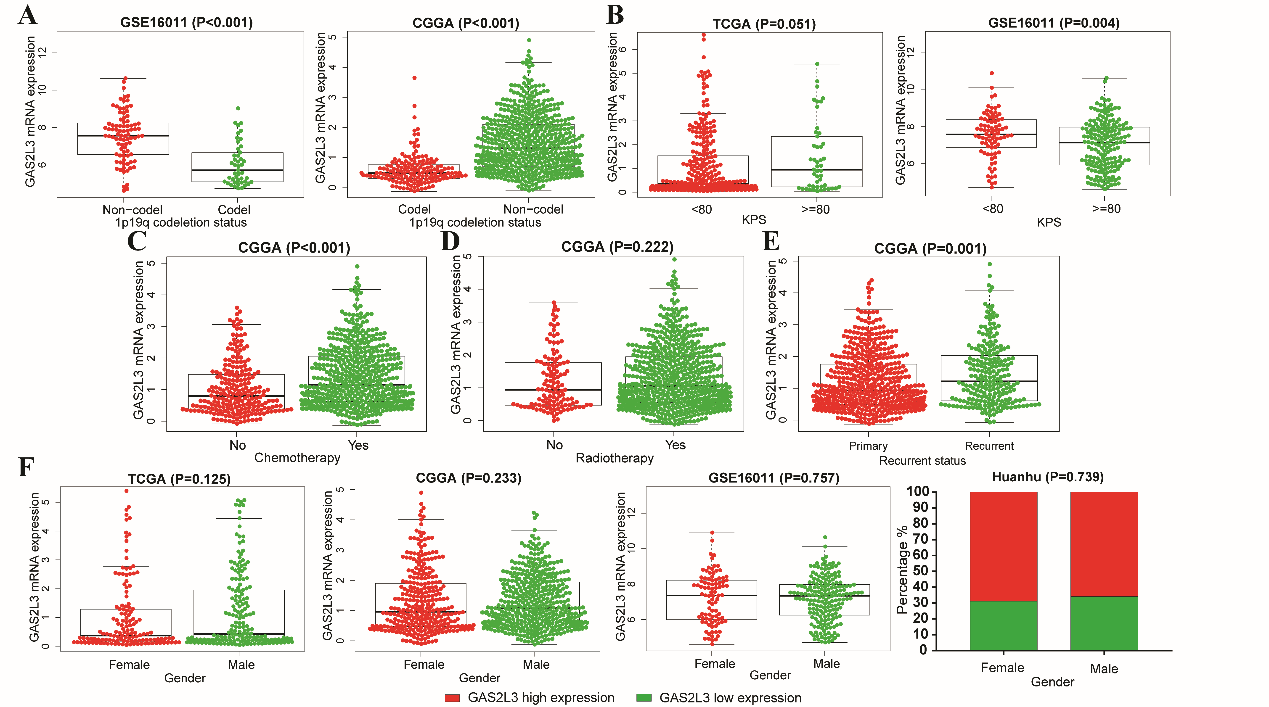


**Figure S2. Associations between GAS2L3 expression and clinicopathologic variables in different datasets.** (A): 1p19q codeletion status, (B): KPS, (C): chemotherapy, (D): radiotherapy, (E): recurrent status, (F): gender (protein level in Huanhu dataset).

**Table S1. The details of patient characteristics in Huanhu dataset**

| **Number** | **Sex** | **Age**  **(year)** | **Histology** | **Grade** | **IDH1 mutation** | **GAS2L3 expression** |
| --- | --- | --- | --- | --- | --- | --- |
| 1 | female | 31 | Astrocytoma | Ⅱ | Yes | high |
| 2 | male | 31 | Astrocytoma | Ⅱ | Yes | low |
| 3 | female | 34 | Astrocytoma | Ⅱ | Yes | low |
| 4 | male | 54 | Astrocytoma | Ⅱ | Yes | high |
| 5 | male | 36 | Astrocytoma | Ⅱ | Yes | high |
| 6 | male | 39 | Astrocytoma | Ⅱ | Yes | high |
| 7 | male | 41 | Astrocytoma | Ⅱ | Yes | high |
| 8 | male | 51 | Astrocytoma | Ⅱ | Yes | high |
| 9 | male | 35 | Astrocytoma | Ⅱ | Yes | high |
| 10 | male | 49 | Astrocytoma | Ⅱ | Yes | low |
| 11 | female | 32 | Astrocytoma | Ⅱ | Yes | high |
| 12 | male | 54 | Astrocytoma | Ⅱ | Yes | high |
| 13 | female | 35 | Astrocytoma | Ⅱ | Yes | high |
| 14 | male | 51 | Astrocytoma | Ⅱ | Yes | low |
| 15 | female | 35 | Astrocytoma | Ⅱ | Yes | high |
| 16 | female | 38 | Astrocytoma | Ⅱ | Yes | low |
| 17 | male | 36 | Astrocytoma | Ⅱ | Yes | high |
| 18 | male | 45 | Astrocytoma | Ⅱ | Yes | high |
| 19 | male | 45 | Astrocytoma | Ⅱ | Yes | low |
| 20 | female | 55 | Astrocytoma | Ⅲ | Yes | high |
| 21 | male | 33 | Astrocytoma | Ⅲ | Yes | low |
| 22 | male | 24 | Astrocytoma | Ⅲ | Yes | high |
| 23 | female | 51 | Astrocytoma | Ⅲ | Yes | low |
| 24 | male | 38 | Astrocytoma | Ⅲ | Yes | low |
| 25 | male | 38 | Astrocytoma | Ⅲ | Yes | high |
| 26 | male | 36 | Astrocytoma | Ⅲ | Yes | low |
| 27 | male | 45 | Astrocytoma | Ⅲ | Yes | low |
| 28 | male | 56 | Astrocytoma | Ⅲ | Yes | low |
| 29 | female | 46 | GBM | Ⅳ | Yes | high |
| 30 | male | 55 | GBM | Ⅳ | Yes | high |
| 31 | male | 44 | GBM | Ⅳ | Yes | low |
| 32 | female | 27 | GBM | Ⅳ | Yes | high |
| 33 | male | 43 | GBM | Ⅳ | Yes | high |
| 34 | female | 50 | GBM | Ⅳ | Yes | high |
| 35 | male | 30 | GBM | Ⅳ | Yes | high |
| 36 | male | 37 | GBM | Ⅳ | Yes | high |
| 37 | female | 40 | GBM | Ⅳ | Yes | high |
| 38 | male | 36 | GBM | Ⅳ | Yes | high |
| 39 | female | 47 | GBM | Ⅳ | Yes | high |
| 40 | female | 35 | GBM | Ⅳ | Yes | high |
| 41 | female | 41 | GBM | Ⅳ | Yes | high |
| 42 | male | 40 | Oligodendroglioma | Ⅱ | Yes | high |
| 43 | male | 48 | Oligodendroglioma | Ⅱ | Yes | high |
| 44 | female | 58 | Oligodendroglioma | Ⅱ | Yes | high |
| 45 | female | 39 | Oligodendroglioma | Ⅱ | Yes | low |
| 46 | male | 48 | Oligodendroglioma | Ⅱ | Yes | high |
| 47 | female | 47 | Oligodendroglioma | Ⅱ | Yes | low |
| 48 | female | 41 | Oligodendroglioma | Ⅱ | Yes | low |
| 49 | female | 50 | Oligodendroglioma | Ⅱ | Yes | low |
| 50 | male | 54 | Oligodendroglioma | Ⅱ | Yes | high |
| 51 | female | 29 | Oligodendroglioma | Ⅱ | Yes | low |
| 52 | male | 46 | Oligodendroglioma | Ⅱ | Yes | low |
| 53 | male | 36 | Oligodendroglioma | Ⅱ | Yes | low |
| 54 | female | 38 | Oligodendroglioma | Ⅱ | Yes | low |
| 55 | female | 21 | Oligodendroglioma | Ⅱ | Yes | low |
| 56 | male | 49 | Oligodendroglioma | Ⅱ | Yes | low |
| 57 | female | 61 | Oligodendroglioma | Ⅱ | Yes | low |
| 58 | male | 42 | Oligodendroglioma | Ⅱ | Yes | low |
| 59 | male | 40 | Oligodendroglioma | Ⅱ | Yes | low |
| 60 | male | 29 | Oligodendroglioma | Ⅱ | Yes | low |
| 61 | male | 45 | Oligodendroglioma | Ⅱ | Yes | low |
| 62 | male | 50 | Oligodendroglioma | Ⅲ | Yes | low |
| 63 | male | 63 | Oligodendroglioma | Ⅲ | Yes | high |
| 64 | male | 42 | Oligodendroglioma | Ⅲ | Yes | high |
| 65 | male | 33 | Oligodendroglioma | Ⅲ | Yes | low |
| 66 | female | 51 | Oligodendroglioma | Ⅲ | Yes | low |
| 67 | female | 36 | Oligodendroglioma | Ⅲ | Yes | low |
| 68 | male | 61 | Oligodendroglioma | Ⅲ | Yes | low |
| 69 | male | 43 | Oligodendroglioma | Ⅲ | Yes | high |
| 70 | female | 48 | Oligodendroglioma | Ⅲ | Yes | low |
| 71 | male | 58 | Oligodendroglioma | Ⅲ | Yes | high |
| 72 | male | 53 | Oligodendroglioma | Ⅲ | Yes | high |
| 73 | female | 55 | Oligodendroglioma | Ⅲ | Yes | low |
| 74 | female | 37 | Oligodendroglioma | Ⅲ | Yes | high |
| 75 | female | 39 | Oligodendroglioma | Ⅲ | Yes | high |
| 76 | female | 51 | Oligodendroglioma | Ⅲ | Yes | low |
| 77 | male | 57 | Oligodendroglioma | Ⅲ | Yes | low |
| 78 | male | 46 | Oligodendroglioma | Ⅲ | Yes | low |
| 79 | male | 55 | Oligodendroglioma | Ⅲ | Yes | high |
| 80 | male | 48 | Oligodendroglioma | Ⅲ | Yes | low |
| 81 | male | 47 | Oligodendroglioma | Ⅲ | Yes | high |
| 82 | female | 58 | Oligodendroglioma | Ⅲ | Yes | low |
| 83 | female | 40 | Oligodendroglioma | Ⅲ | Yes | high |
| 84 | male | 44 | Oligodendroglioma | Ⅲ | Yes | high |
| 85 | male | 53 | Oligodendroglioma | Ⅲ | Yes | high |
| 86 | female | 50 | Oligodendroglioma | Ⅲ | Yes | high |
| 87 | female | 35 | Oligodendroglioma | Ⅲ | Yes | high |
| 88 | female | 66 | GBM | Ⅳ | No | high |
| 89 | male | 60 | GBM | Ⅳ | No | high |
| 90 | male | 49 | GBM | Ⅳ | No | high |
| 91 | female | 65 | GBM | Ⅳ | No | high |
| 92 | female | 40 | GBM | Ⅳ | No | high |
| 93 | female | 72 | GBM | Ⅳ | No | high |
| 94 | male | 42 | GBM | Ⅳ | No | high |
| 95 | female | 40 | GBM | Ⅳ | No | high |
| 96 | male | 61 | GBM | Ⅳ | No | high |
| 97 | male | 65 | GBM | Ⅳ | No | high |
| 98 | male | 44 | GBM | Ⅳ | No | high |
| 99 | male | 64 | GBM | Ⅳ | No | low |
| 100 | female | 62 | GBM | Ⅳ | No | high |
| 101 | male | 48 | GBM | Ⅳ | No | high |
| 102 | female | 67 | GBM | Ⅳ | No | high |
| 103 | male | 55 | GBM | Ⅳ | No | high |
| 104 | male | 54 | GBM | Ⅳ | No | high |
| 105 | male | 64 | GBM | Ⅳ | No | high |
| 106 | male | 61 | GBM | Ⅳ | No | high |
| 107 | female | 82 | GBM | Ⅳ | No | high |
| 108 | female | 41 | GBM | Ⅳ | No | high |
| 109 | female | 60 | GBM | Ⅳ | No | high |
| 110 | male | 72 | GBM | Ⅳ | No | high |
| 111 | female | 39 | GBM | Ⅳ | No | high |
| 112 | male | 50 | GBM | Ⅳ | No | high |
| 113 | male | 60 | GBM | Ⅳ | No | high |
| 114 | female | 67 | GBM | Ⅳ | No | high |
| 115 | female | 67 | GBM | Ⅳ | No | high |
| 116 | female | 59 | GBM | Ⅳ | No | high |
| 117 | male | 39 | GBM | Ⅳ | No | high |
| 118 | female | 59 | GBM | Ⅳ | No | high |
| 119 | male | 64 | GBM | Ⅳ | No | high |
| 120 | male | 52 | GBM | Ⅳ | No | high |
| 121 | male | 59 | GBM | Ⅳ | No | high |
| 122 | male | 51 | GBM | Ⅳ | No | high |
| 123 | male | 65 | GBM | Ⅳ | No | high |
| 124 | male | 17 | GBM | Ⅳ | No | high |
| 125 | male | 66 | GBM | Ⅳ | No | high |
| 126 | male | 68 | GBM | Ⅳ | No | high |
| 127 | male | 69 | GBM | Ⅳ | No | high |

**Table S2. Gene sets enriched in high GAS2L3 expression phenotype in TCGA.**

| **Dataset** | **Gene set** | **NES** | **NOM p-val** | **FDR q-val** |
| --- | --- | --- | --- | --- |
| **KEGG** | KEGG_LEUKOCYTE_TRANSENDOTHELIAL_MIGRATION | 1.97 | 0 | 0.0104 |
|  | KEGG_T_CELL_RECEPTOR_SIGNALING_PATHWAY | 1.55 | 0.0417 | 0.0800 |
|  | KEGG_TOLL_LIKE_RECEPTOR_SIGNALING_PATHWAY | 1.61 | 0.0366 | 0.0644 |
|  | KEGG_PRIMARY_IMMUNODEFICIENCY | 1.67 | 0.0356 | 0.0502 |
|  | KEGG_CYTOKINE_CYTOKINE_RECEPTOR_INTERACTION | 1.68 | 0.0343 | 0.0509 |
|  | KEGG_FC_GAMMA_R_MEDIATED_PHAGOCYTOSIS | 1.60 | 0.0273 | 0.0679 |
|  | KEGG_JAK_STAT_SIGNALING_PATHWAY | 1.64 | 0.0207 | 0.0603 |
|  | KEGG_NATURAL_KILLER_CELL_MEDIATED_CYTOTOXICITY | 1.68 | 0.0204 | 0.0524 |
|  | KEGG_ANTIGEN_PROCESSING_AND_PRESENTATION | 1.74 | 0.0201 | 0.0383 |
| GO | GO_REGULATION_OF_INNATE_IMMUNE_RESPONSE | 1.91 | 0 | 0.0227 |
|  | GO_REGULATION_OF_LYMPHOCYTE_MIGRATION | 1.93 | 0 | 0.0230 |
|  | GO_REGULATION_OF_B_CELL_MEDIATED_IMMUNITY | 1.94 | 0.0185 | 0.0231 |
|  | GO_IMMUNE_RESPONSE_TO_TUMOR_CELL | 1.94 | 0.0217 | 0.0233 |
|  | GO_POSITIVE_REGULATION_OF_ADAPTIVE_IMMUNE_RESPONSE | 1.88 | 0 | 0.0250 |
|  | GO_RESPONSE_TO_TUMOR_NECROSIS_FACTOR | 2.03 | 0 | 0.0270 |
|  | GO_MYELOID_LEUKOCYTE_DIFFERENTIATION | 1.85 | 0 | 0.0286 |
|  | GO_T_CELL_MEDIATED_IMMUNITY | 1.84 | 0 | 0.0293 |
|  | GO_REGULATION_OF_IMMUNOGLOBULIN_PRODUCTION | 2.02 | 0 | 0.0296 |
|  | GO_RECEPTOR_SIGNALING_PATHWAY_VIA_STAT | 1.83 | 0.0217 | 0.0296 |
|  | GO_POSITIVE_REGULATION_OF NF_KAPPAB_SIGNALING | 1.97 | 0 | 0.0257 |

NES: normalized enrichment score; NOM: nominal; FDR: false discovery rate. Gene sets with NOM P-value <0.05 and FDR q-value <0.25 were considered as significantly enriched.
